# Aberrant promoter methylation of hOGG1 may be associated with increased risk of non-small cell lung cancer

**DOI:** 10.18632/oncotarget.14177

**Published:** 2016-12-26

**Authors:** Hualong Qin, Jianjie Zhu, Yuanyuan Zeng, Wenwen Du, Dan Shen, Zhe Lei, Qian Qian, Jian-an Huang, Zeyi Liu

**Affiliations:** ^1^ Department of Cardiothoracic Surgery, The First Affiliated Hospital of Soochow University, Suzhou, 215006, China; ^2^ Department of Respiratory Medicine, The First Affiliated Hospital of Soochow University, Suzhou, 215006, China; ^3^ Institute of Respiratory Diseases, Soochow University, Suzhou, 215006, China; ^4^ Laboratory of Cancer Molecular Genetics, Medical College of Soochow University, Suzhou 215123, China; ^5^ Division of Allergy & Immunology, Department of Medicine, National Jewish Health, Denver, CO 80206, USA

**Keywords:** non-small cell lung cancer, base excision repair, hOGG1, SNP, methylation

## Abstract

DNA methylation may epigenetically inactivate tumor suppressor genes in NSCLC. As the human 8-oxoguanine DNA glycosylase (*hOGG1)* gene promoter is frequently methylated in NSCLC, we evaluated whether genetic or epigenetic alterations of *hOGG1* are associated with increased risk of non-small cell lung cancer. Three *hOGG1* haplotype-tagging SNPs (htSNP) were genotyped in PCR-restriction fragment length polymorphism assays, and one htSNP was genotyped in a PCR-single-strand conformation polymorphism assay in case-control studies of 217 NSCLC patients and 226 healthy controls. The methylation profiles of peripheral blood mononuclear cell specimens from 121 NSCLC patients and 121 controls were determined through methylation-specific PCR of *hOGG1*. No differences in allele or genotype frequencies between NSCLC patients and controls were observed at any of the four polymorphic sites (rs159153, rs125701, rs1052133, and rs293795). However, *hOGG1* methylation-positive carriers had a 2.25-fold greater risk of developing NSCLC (adjusted odds ratio: 2.247; 95% confidence interval: 1.067-4.734; *P* = 0.03) than methylation-free subjects. Furthermore, the demethylating agent 5-aza-2’-deoxycytidine restored *hOGG1* expression in NSCLC cell lines. These data provide strong evidence of an association between peripheral blood mononuclear cell *hOGG1* methylation and the risk of NSCLC in a Chinese population.

## INTRODUCTION

Lung cancer is the leading cause of cancer-related death in China and worldwide [1, 2]. Non-small cell lung cancer (NSCLC) accounts for approximately 85% of lung cancer cases. Despite improvements in cancer treatment, the five-year survival rate for patients with this cancer is still less than 10%. However, if patients are diagnosed with cancer and receive surgery at an early stage, the five-year survival rate can be as high as 55-80% [3]. It has been suggested that NSCLC could result from the accumulation of multiple genetic or epigenetic changes, including DNA methylation, histone acetylation [4], and dysregulation of microRNA [5, 6]. Increased understanding of the mechanisms of tumor development and progression will be important for early detection, prevention, and targeted treatment for NSCLC.

There is frequently functional overlap in DNA repair pathways, guaranteeing genomic stability and challenging the concept that various lesions in the mammalian genome are repaired by different mechanisms. This is particularly true for oxidatively damaged DNA (e.g., oxidized bases) [7], which may result from ionizing radiation or oxidant exposure, as well as from normal cellular metabolism. Oxidized bases are cytotoxic and mutagenic, and several lines of evidence indicate that they may contribute to aging, neurodegeneration, and cancer [8]. Base excision repair (BER) is an important DNA repair pathway for base damage and single-strand breaks caused by X-rays, oxygen radicals, or alkylating agents [9].

One of the enzymes in the DNA BER pathway is 8-oxoguanine DNA glycosylase (OGG1). This DNA repair glycosylase is localized to both the nucleus and mitochondria, and is the main enzyme that identifies and excises 8-oxoG lesions that produce G:C to T:A transversions [10–12]. The human OGG1 gene (*hOGG1*) is found on chromosome 3p26.2, which is one of the most frequent regions of genomic deletion and contains potential tumor suppressor genes for various types of tumors (e.g., NSCLC) [13]. *hOGG1* is a polygenetic gene that plays a role in several disease pathways, including various cancers [14–16]. Genetic variants in *hOGG1*, including single-nucleotide polymorphisms (SNPs), may affect the expression and function of the OGG1 protein, thus contributing to the risk of NSCLC and influencing the prognosis of patients. Ser326Cys is the most highly investigated *hOGG1* SNP, and several studies have suggested that the Cys326 allele is associated with increased risk of lung cancer [17–19]. However, the function of Ser326 remains controversial [20–23].

It is well established that gene expression can be epigenetically regulated via changes in DNA methylation [24–26], which frequently occur in CpG islands around the 5’-untranslated regions (5’-UTRs) of genes [27]. In particular, site-specific DNA methylation alterations in CpG islands, including the hypomethylation of oncogenes and the hypermethylation of tumor suppressor genes, may be crucial promoters of cancer progression [24, 28]. Our previous studies support the notion that DNA methylation could epigenetically inactivate tumor suppressor genes on the short arm of chromosome 3p in NSCLC [13] and thus increase the risk of non-small cell lung cancer [29]. Hence, we hypothesized that hypermethylation of the *hOGG1* promoter in peripheral blood mononuclear cells (PBMCs) could affect *hOGG1* mRNA expression and increase the risk of NSCLC.

To verify the relationship between the genetic variants and susceptibility to NSCLC comprehensively, we first genotyped four *hOGG1* SNPs (htSNP) using PCR-restriction fragment length polymorphism analysis, and one htSNP using PCR-single-strand conformation polymorphism analysis. The four htSNPs, including two in the 5′ flanking region, one in an intronic region, and one in the 3′ flanking region of the *hOGG1* gene, appropriately captured all of the common haplotype blocks reconstructed in HapMap Phase II data. In addition, the methylation status of the promoter region was detected by methylation-specific PCR (MSP) in Chinese population-based case-control studies.

## RESULTS

### Characteristics of patients with lung cancer and controls

The general clinical characteristics of 217 NSCLC patients and 226 cancer-free controls are listed in Table 1. The NSCLC patients and cancer-free controls were comparable with regard to the distribution of gender and age (all *P* > 0.05).

**Table 1:** Characteristics of the case-control study subjects

Variable	NSCLC Patients	Controls
	N=217, n (%)	N=226, n (%)
Age (yrs)		
<50	23 (10.6)	73 (32.3)
50-69	145 (66.8)	104 (46.0)
≥70	49 (22.6)	49 (21.7)
Mean ± SD	62.10±9.76	55.26±16.06
Sex		
Male	154 (71.0)	134 (59.2)
Female	63 (29.0)	92 (40.8)
TNM stage		
I	58 (26.7)	
II	46 (21.2)	
III	39 (17.9)	
IV	74 (34.1)	
Histological type		
Adenocarcinoma	78 (35.9)	
Squamous carcinoma	99 (45.6)	
Others	40 (18.5)	

### *hOGG1* genotypes and the risk of lung cancer

**Figure 1:**
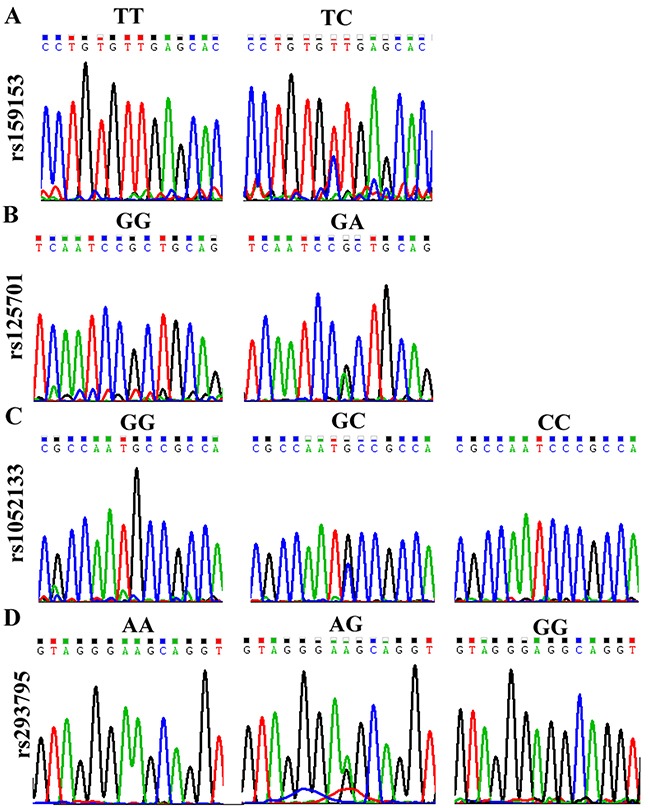
DNA sequencing results of a typical genotyping experiment **A**. rs159153, **B**. rs125701, **C**. rs159153, and **D**. rs293795.

To determine whether any of the four promoter variants in *hOGG1* (rs159153, rs125701, rs1052133, and rs293795) modify the risk of lung cancer, we genotyped participants for these four SNPs. The genotype frequencies of these polymorphisms were in Hardy-Weinberg equilibrium in the controls. To further validate these results, we used a sequencing method to genotype these SNPs; representative genotyping results are presented in Figure 1. No significant differences were observed in the allele and genotype frequencies of any of these four polymorphic sites between NSCLC patients and controls (Table 2). After stratifying subjects by age, gender, smoking history and histology, we did not observe any association of these polymorphisms with lung cancer risk in either group (Table 3).

**Table 2:** Genotype and allele frequencies of haplotype tagging SNPs among NSCLC cases and controls, and associations with the risk of NSCLC

		Cases	Controls	*p*	*p* for HWE	OR(95%CI)	*p*
		N=217, n(%)	N=226, n(%)				
rs159153							
Genotype	TT	187(86.2)	197(87.2)	0.758	0.588	1.00	
	TC	30(13.8)	29(12.8)			1.090(0.630-1.886)	0.758
	CC	0(0.0)	0(0.0)				
	TC/CC	30(13.8)	29(12.8)			1.090(0.630-1.886)	0.758
Allele	T	404(93.1)	423(93.6)			1.00	
	C	30(6.9)	29(6.4)			1.083(0.639-1.837)	0.767
rs125701							
Genotype	GG	200(92.2)	203(89.8)	0.390	0.723	1.00	
	GA	17(7.8)	23(10.2)			1.333(0.691-2.570)	0.390
	AA	0(0.0)	0(0.0)				
	GA/AA	17(7.8)	23(10.2)			1.333(0.691-2.570)	0.390
Allele	G	417(96.1)	429(94.9)			1.00	
	A	17(3.9)	23(5.1)			1.315(0.693-2.497)	0.401
rs1052133							
Genotype	GG	59(27.2)	72(31.9)	0.391	0.121	1.00	
	GC	121(55.8)	124(54.9)			1.191(0.778-1.823)	0.421
	CC	37(17.0)	30(13.2)			1.505(0.833-2.720)	0.175
	GC/CC	158(72.8)	154(68.1)			1.252(0.831-1.886)	0.282
Allele	G	239(55.1)	268(59.3)			1.00	
	C	195(44.9)	184(40.7)			1.188(0.910-1.551)	0.204
rs293795							
Genotype	TT	199(91.7)	210(92.9)	0.378	0.458	1.00	
	TC	14(6.5)	15(5.8)			0.985(0.464-2.093)	0.968
	CC	4(1.8)	1(1.3)			4.221(0.468-38.091)	0.163
	TT/CC	18(8.3)	16(7.1)			1.187(0.589-2.393)	0.631
Allele	T	412(94.9)	433(95.8)			1.00	
	C	22(5.1)	19(4.2)			1.217(0.649-2.281)	0.540

*p* value for Chi-square analysis or Fisher's exact test. HWE, Hardy-Weinberg equilibrium

**Table 3:** Associations between genotypes and clinical characteristics of NSCLC

	Genotypes			*p*
	wt/wt	wt/mt	mt/mt	
**rs159153**				
Histology				
Adenocarcinoma	69	8	1	
Squamous carcinoma	80	18	1	
Others	38	2	0	0.221
TNM Stage				
I+II	92	11	1	
III+IV	95	17	1	0.618
**rs125701**				
Histology				
Adenocarcinoma	69	9	0	
Squamous carcinoma	93	6	0	
Others	37	3	0	0.414
TNM Stage				
I+II	99	5	0	
III+IV	101	12	0	0.111
**rs1052133**				
Histology				
Adenocarcinoma	23	47	15	
Squamous carcinoma	23	54	15	
Others	13	20	7	0.908
TNM Stage				
I+II	36	53	15	
III+IV	23	68	22	0.058
**rs293795**				
Histology				
Adenocarcinoma	68	8	2	
Squamous carcinoma	91	6	2	
Others	40	0	0	0.214
TNM Stage				
I+II	95	8	1	
III+IV	104	6	3	0.516

wt/wt represents a wild-type homozygote; wt/mt represents a heterozygote; mt/mt represents a homozygous mutation. *p* value for Chi-square analysis or Fisher's exact test.

### *hOGG1* haplotypes and risk of lung cancer

**Figure 2:**
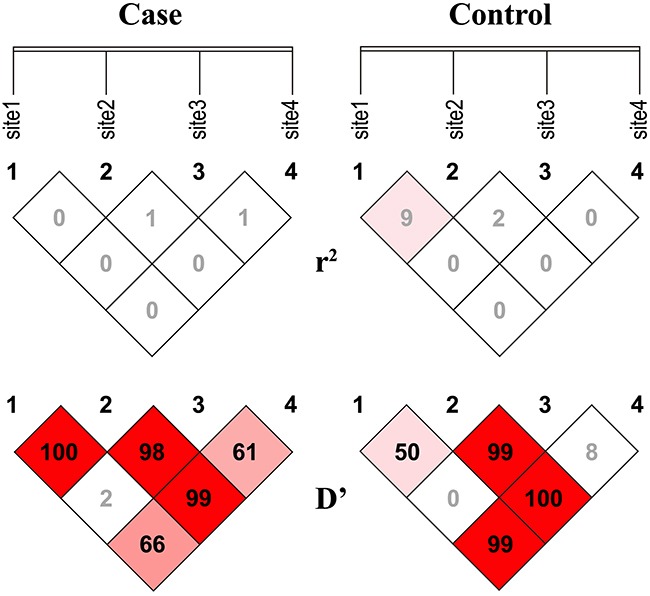
LD maps of the four htSNPs LD maps of the four htSNPs of hOGG1 for NSCLC cases and controls were generated by the SHEsis program. For each htSNP, D′ and pairwise r2 values are shown in diamonds for the two groups (NSCLC patients and healthy controls). Sites 1-4 represent rs159153, rs125701, rs159153, and rs293795, respectively.

Association studies based on haplotypes of multiple markers have significantly greater power than genotypes at single markers for mapping and characterizing disease-causing genes [30]. Thus, we sought to assess whether various haplotypes consisting of the four SNPs of the *hOGG1* promoter (rs159153, rs125701, rs1052133, rs293795) were associated with the risk of lung cancer. As shown in Figure 2, linkage disequilibrium (LD) analysis revealed that the D′ values of rs159153 with rs125701 and of rs159153 with rs293795 were > 0.80. In contrast, the D′ values of rs159153 with rs125701 and of rs159153 with rs293795 were < 0.80 in the controls (D′ =1.0 vs. D′ =0.5, D′ =0.99 vs. D′ =0.66). Accordingly, four-SNP haplotypes (rs159153, rs125701, rs1052133, and rs293795) and two-SNP haplotypes (rs159153, rs125701, and rs159153, rs293795) were reconstructed according to the genotyping data from the NSCLC patients and controls. Using haplotypes with frequencies of > 0.02 for further analysis, we found that no haplotype was associated with significantly greater risk of lung cancer (Tables 4 and 5).

**Table 4:** Four-marker haplotype frequencies in NSCLC patients and control subjects

Haplotype	Allele combination	Frequencies*	Chi-square	OR(95%CI)	*p*
Hap1	TGGT	50.4/50.3	0.042	0.96(0.67-1.39)	0.838
Hap2	TGCT	36.9/37.7	0.118	0.94(0.64-1.36)	0.731
Hap3	CGGT	4.1/3.0	0.362	1.35(0.51-3.58)	0.547
Hap4	TGGC	3.4/1.6	1.442	2.08(0.62-7.02)	0.230
Hap5	CGCT	3.0/3.2	0.040	0.90(0.32-2.54)	0.842

* Haplotype frequencies (%) in the NSCLC and control groups.

**Table 5:** Two-marker haplotype frequencies in NSCLC patients and control subjects

Haplotype	Allele combination	Frequencies*	Chi-square	OR(95%CI)	*p*
Site1&site2					
Hap1	T G	91.5/90.8	0.149	0.87(0.42-1.78)	0.699
Hap2	C G	7.3/6.3	0.149	1.15(0.56-2.36)	0.699
Site1&site4					
Hap1	T T	88.6/89.3	0.044	0.94(0.53-1.67)	0.835
Hap2	C T	7.2/7.9	0.080	0.91(0.46-1.79)	0.778
Hap3	T C	4.2/2.9	0.575	1.46(0.55-3.93)	0.448

* Haplotype frequencies (%) in the NSCLC and control groups. Site 1, site 2, and site 4 represent rs159153, rs125701, and rs293795, respectively.

### The frequency of *hOGG1* promoter methylation and NSCLC risk

**Figure 3:**
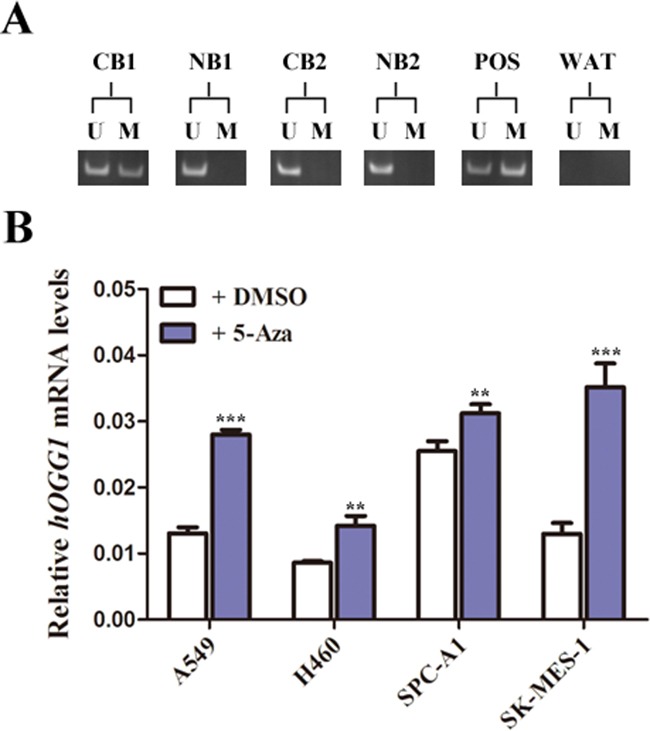
Methylation status analysis of hOGG1 in NSCLC **A**. A schematic presentation of methylation analysis of the hOGG1 gene promoter, based on MSP of cancer patients’ blood (CB) and normal blood (NB). POS, positive methylation; water, negative control. The presence of a PCR product in lane U indicates an unmethylated status, while M indicates a methylated status. **B**. NSCLC cell lines, untreated or treated with the demethylation agent 5-aza. The cells were grown to 80% confluency and were harvested on day 7.

Our previous work indicated that DNA methylation could epigenetically inactivate tumor suppressor genes in NSCLC. Moreover, the *hOGG1* gene promoter is frequently methylated in NSCLC [13, 29]. Therefore, we used methylation-specific PCR (MSP) to determine the methylation profiles for *hOGG1* in PBMC specimens from 121 NSCLC patients and 121 controls. Representative bands from MSP analysis are shown in Figure 3A. Among NSCLC patients, the frequency of *hOGG1* promoter methylation was 19.83%. In contrast, a lower frequency of *hOGG1* methylation (9.92%) was detected in controls (Table 6). To determine whether positivity for *hOGG1* methylation increases the risk of NSCLC, we performed logistic regression analysis to assess the odds ratios (ORs) for NSCLC in the paired cohort. The risk of NSCLC was significantly greater among those with methylation of *hOGG1* than among those who were methylation-free (2.25-fold, *P* < 0.05, Table 6).

**Table 6:** Frequency of promoter methylation for *hOGG1* in NSCLC and matched non-cancerous tissue

*hOGG1* Methylation Status	CasesN=121, n (%)	ControlsN=121, n (%)	*p*	OR (95%CI)
U	97 (80.17)	109 (90.08)		1.00
M	24 (19.83)	12 (9.92)	0.030	2.247(1.067-4.734)

*p* value for Chi-square analysis

### Differential expression of *hOGG1* mRNA is associated with DNA methylation in NSCLC cell lines

To determine whether methylation of the *hOGG1* gene promoter is an alternative mechanism of inactivating *hOGG1* mRNA expression, we evaluated mRNA expression after treating NSCLC cell lines with the demethylating agent 5-aza-2’-deoxycytidine (5-Aza) (Figure 3B). *hOGG1* mRNA expression was elevated in 5-aza-treated NSCLC cell lines (A549, H460, SPC-A1, 95D, and SK-MES-1), suggesting that *hOGG1* mRNA expression is associated with DNA methylation.

## DISCUSSION

Epidemiological evidence has documented that lung cancer incidence and mortality have increased markedly over the past decade in China [2]. One promising treatment approach for lung cancer is the identification of lung cancer-specific biomarkers at an early stage. NSCLC could result from the accumulation of multiple genetic and/or epigenetic aberrations, and DNA methylation could epigenetically inactivate tumor suppressor genes in NSCLC [13]. The *de novo* methylation of CpG islands within the promoters of tumor suppressor genes is one of the most frequently acquired epigenetic changes during the pathogenesis of lung cancer, and is usually associated with the transcriptional downregulation of the affected genes. The analysis of DNA methylation patterns in the sputum, bronchial fluid, plasma, or serum could become a powerful tool for the accurate and early diagnosis of lung cancer, with unparalleled specificity and sensitivity [29, 31, 32].

Genetic variants of *hOGG1*, including SNPs, may affect the expression and function of the OGG1 protein, thus contributing to the risk of NSCLC and influencing the prognosis of patients [33, 34]. In an effort to validate the association of *hOGG1* SNPs or haplotypes with NSCLC risk, we analyzed tagging SNPs and haplotypes to capture the genetic variants of *hOGG1* in the Chinese population comprehensively. There were no significant differences in allele and genotype frequencies or even haplotypes between NSCLC patients and controls. This suggests that no individual *hOGG1* SNP examined in this study is associated with NSCLC risk.

Given the above observations, additional studies were needed to determine whether aberrant promoter methylation of *hOGG1* increases the risk of developing NSCLC. First, to determine whether methylation of the *hOGG1* gene promoter is an alternative mechanism of inactivating *hOGG1* mRNA expression, we evaluated *hOGG1* mRNA expression after treating NSCLC cell lines with the demethylating agent 5-Aza. *hOGG1* mRNA expression increased in NSCLC cell lines after treatment with 5-Aza, suggesting that *hOGG1* mRNA expression is associated with DNA methylation. Furthermore, using MSP, we found that the frequency of *hOGG1* methylation was higher in PBMCs from NSCLC patients than in those from controls. Although DNA methylation is frequent in NSCLC PBMCs [35], we cannot exclude the possibility that the detected methylation was derived from circulating tumor cells in the peripheral blood. Our data indicated that positive carriers of *hOGG1* methylation had a 2.25-fold greater risk of developing NSCLC than methylation-free subjects in the Chinese population. Thus, our combined analysis using genetics and epigenetics revealed that the methylation status of *hOGG1* in PBMCs could be a marker for NSCLC.

In conclusion, we provide strong evidence of an association between the methylation of *hOGG1* and the risk of NSCLC. This supports the notion that peripheral lymphocytes could be used as surrogates for bronchial epithelial cells in screenings for altered DNA methylation in the development and progression of lung cancer [29, 38]. Considering that treatment with demethylating agents can restore gene expression and inactivate *hOGG1* [39], the use of such agents may be the ideal way to reduce the risk of developing NSCLC among individuals with *hOGG1* hypermethylation. A better understanding of the methylation status of BER genes may enhance cancer prevention and therapeutic interventions.

## MATERIALS AND METHODS

### Patients and blood samples

In total, 217 blood specimens were aquired after informed consent was received from patients who had been diagnosed with primary NSCLC in the First Affiliated Hospital of Soochow University between January 2003 and March 2013. The pathological stages of NSCLC patients were determined according to the Revised International System for Staging Lung Cancer. None of the NSCLC patients had received either radiotherapy or chemotherapy before blood sampling. In addition, 226 control samples were collected from individuals with no history of cancer; these participants were randomly selected from the same geographic region, and their age range was similar to that of the cancer patients. PBMCs were isolated by centrifugation at 3,500 rpm for 20 min after blood sampling [29]. Genomic DNA was isolated from NSCLC cell lines and blood samples through a standard proteinase K digestion and phenol-chloroform extraction. This study was approved by the Ethics Committee of the First Affiliated Hospital of Soochow University. A standardized questionnaire was used to collect data on age, sex, and smoking history.

### Cell culture and drug treatment

Human lung carcinoma cell lines (A549, SPC-A-1, NCI-H460, and SK-MES-1) were purchased from the Cell Bank of the Chinese Academy of Sciences (Shanghai, China). A549, SPC-A-1, and NCI-H460 cells were seeded and grown in RPMI 1640 medium (HyClone, South Logan, UT, USA) with 10% heat-inactivated fetal bovine serum (Gibco, Carlsbad, CA, USA). SK-MES-1 cells were cultured in MEM (Gibco) with 10% fetal bovine serum (Gibco), L-glutamine, and antibiotics (Invitrogen, Carlsbad, CA, USA). All cells were cultured in a humidified incubator containing 5% CO_2_ at a temperature of 37°C. 5-aza-2’-deoxycytidine (Sigma–Aldrich, St. Louis, MO, USA) was used as a demethylating agent to treat the cells. The drug treatment protocol was described previously by us [29].

### Tagging SNP selection

**Figure 4:**
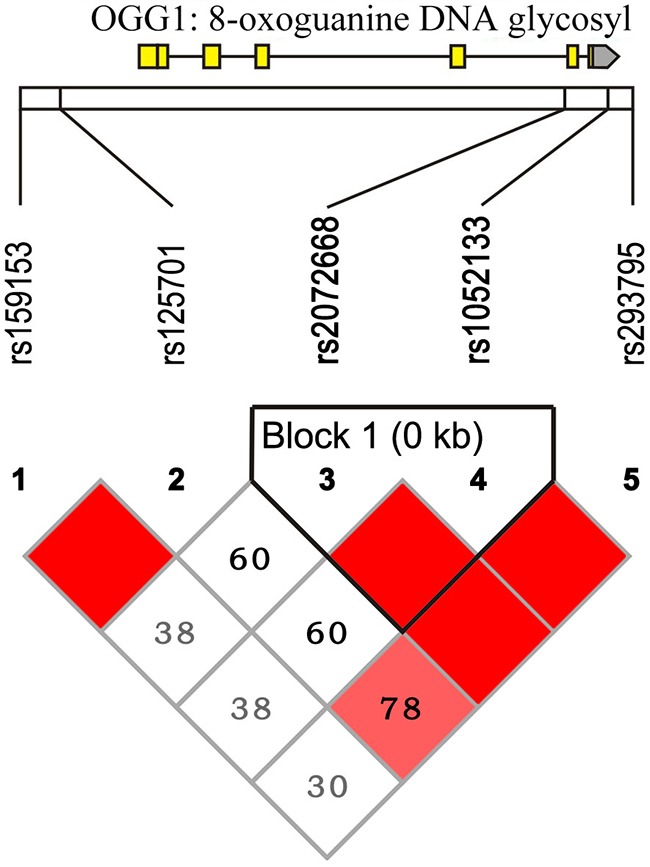
Pairwise LD between four htSNPs of the hOGG1 gene The value in each diamond indicates the pairwise correlation between the tagging SNPs (measured as r2) located at the upper left and right sides of the diamond. The shading with a red-to-white gradient reflects higher to lower LD values (measured as D′).

HapMap SNP Phase II data were used to determine the frequency of SNPs among Han Chinese (CHB), and five SNPs were obtained from a 15-kb region of *hOGG1*. One haplotype block was reconstructed from these five SNPs in the Haploview program [36]. The htSNP selection was also made in the Haploview program, through the implementation of a method proposed in our previous study [30]. A SNP was considered for inclusion in the set of htSNPs if the r^2^ value for its LD with at least one of the other SNPs was greater than a prespecified threshold. In our selection, only SNPs with minor allele frequencies greater than 10% were considered, and the threshold of pairwise LD was set as r^2^ = 0.8. A total of four htSNPs within one block were selected among five SNPs considered across *hOGG1*. The LD map of these four htSNPs is shown in Figure 4, and further information is provided in Supplementary Table 1.

### Genotyping

Genomic DNA from blood specimens was isolated by standard proteinase K digestion and phenol-chloroform extraction. The four *hOGG1* htSNPs were amplified by PCR. The sequences and annealing temperatures of the PCR primers are reported in Supplementary Table 2. The PCR reactions were carried out in a total volume of 25 μL, containing 50 to 100 ng of genomic DNA, one unit of ExTaq DNA polymerase (Takara, Japan), 0.2 μmol/L of each primer, 1× Ex Taq Buffer (Mg^2+^ Plus), and 0.25 mmol/L of each deoxynucleotide triphosphate.

The htSNPs were genotyped by restriction fragment length polymorphism analysis with restriction endonucleases (Supplementary Table 2). The different alleles were identified on a 2.5% agarose gel and visualized with ethidium bromide. One htSNP (rs293795) was genotyped by single-strand conformation polymorphism analysis due to the lack of a restriction endonuclease. For this procedure, the PCR products were mixed at a 1:1 ratio with loading buffer (95% formamide, 0.05% xylene cyanol, and 0.05% bromophenol blue), denatured at 95°C for 5 min and cooled on ice for 2 min. Electrophoresis was performed in 8% nondenaturing polyacrylamide gels at a constant 20 W for 5 h in 1× Tris-borate-EDTA running buffer, with the gel temperature maintained at 7°C. Ethidium bromide staining was used for the detection of single-stranded DNA in the polyacrylamide gels.

### LD and haplotype analysis

Pairwise measures of LD (the Lewontin coefficient [D′] and the squared correlation coefficient [r^2^]) between the genotyped SNPs were calculated with the Haploview program. The frequencies of individual haplotypes were estimated from the genotype data through the SAS 9.1.3 PROC HAPLOTYPE and SHEsis software programs [37], which reconstruct haplotypes through an expectation-maximization algorithm and a Full-Precise-Iteration algorithm, respectively. Haplotypes with a frequency of less than 0.05 were not considered in the analysis. Logistic regression analysis was performed with SAS PROC LOGISTIC to estimate the ORs and 95% confidence intervals (95% CIs) of individual SNPs or haplotypes, with adjustment for age, sex, and smoking status.

### Bisulfite modification

For methylation analysis, we collected 121 blood samples from NSCLC patients and 121 blood samples from control subjects. Genomic DNA was treated with sodium bisulfite before methylation analysis, according to the protocol of the CpGenome™ Fast DNA Modification Kit (Chemicon International, Inc.). The procedure for bisulfite modification was described in our previously published report [13]. Briefly, 1.0 μg of DNA in 100 μL of water was denatured for 10 min at 37°C with the addition of 7 μL of freshly prepared 3M NaOH. Then, 550 μL of freshly prepared DNA Modification Reagent (pH 5.0) was added and well-mixed, and the mixture was incubated at 55°C for 20 h. After adding 750 μL of Binding Buffer, we purified the modified DNA with spin columns. Desulphonation was completed on the column by the addition of 50 μL of freshly prepared 20 mM NaOH/90% EtOH. Finally, DNA was eluted with 30-45 μL of Elution Buffer and stored at -20°C until the analysis.

### Methylation-specific PCR

DNA methylation was determined by MSP, as described elsewhere. MSP can differentiate methylated alleles from unmethylated alleles of a gene-of-interest, after samples have been treated with sodium bisulfite. The primer sequences for MSP analysis of *hOGG1* were reported in a previous study [13]. Briefly, the PCR cycling conditions consisted of an initial denaturation at 95°C for 5 min, followed by 35 cycles of 94°C for 30 sec, various annealing temperatures for 45 sec, and 72°C for 1 min and 15 sec. The expected PCR products were revealed by electrophoresis and visualized with ethidium bromide staining. SPC-A-1 and A549 NSCLC cell lines were used as positive controls for *hOGG1*, while distilled water and unbisulfited DNA served as negative controls.

### Statistical analysis

We used a two-sided χ^2^ test or an independent-samples *t-*test to compare the differences in gender, age, and smoking status between the NSCLC cases and controls. Hardy-Weinberg equilibrium analysis for genotype distribution in controls was carried out by a χ^2^ goodness-of-fit test. Differences in genotype and allele frequencies between cases and controls were determined with a χ^2^ test. Logistic regression was performed to assess the ORs and 95% CIs, which were adjusted for gender, age, and smoking status. All statistical analyses were performed with SAS 9.1.3., and the statistical significance cutoff was *P* < 0.05.

## SUPPLEMENTARY MATERIALS FIGURES AND TABLES


