# Supplementary material for: Aberrant promoter methylation of hOGG1 may be associated with increased risk of non-small cell lung cancer
Source: Oncotarget. 2016 Dec 26;8(5):8330–41. doi: 10.18632/oncotarget.14177 (PMC5352404; doi:10.18632/oncotarget.14177)
Supplement: Supplementary file 1 [file oncotarget-08-8330-s001.pdf]

Aberrant promoter methylation of *hOGG1* may be associated with increased risk of non-small cell lung cancer

SUPPLEMENTARY TABLES

Supplementary Table 1: Information on four htSNPs of the *hOGG1* gene in the Chinese population*

SNP	ID	Chromosome position	Location in gene	Base change	Minor allele (frequency)
SNP1	rs159153	100874968	5'-flanking	T > C	C(0.089)
SNP2	rs125701	100889547	5'-flanking	G > A	G(0.028)
SNP3	rs1052133	100904795	exon-7	G / C	C(0.494)
SNP4	rs293795	100914135	3'-flanking	A > G	A(0.061)

*SNP positions and minor allele frequencies are based on the NCBI dbSNP Build 129 (http://www.ncbi.nlm.nih.gov/snp/).

Supplementary Table 2: Primers and restriction endonucleases used to genotype *hOGG1*

htSNP ID	Primers			Restriction endonucleases		
	Primer sequences (5′-3′)	Tm (ºC)	Product length (bp)	Restriction endonuclease	Incubation temperature (ºC)	Specific allele (position)
rs159153	FP: GATACAAAGGGAGGTTCTG	56	180	Bpu1102 I	37	C(142)
	RP: CAAGGATTACGCAAAGC					
rs125701	FP: CTTCTTTCCAGCTCCACCGA	58	296	MspA1I	37	G(227)
	RP: GTTGGGATTACAGGCGTGAG					
rs1052133	FP: GGGCCCAAGCGGTGAGT	60	336	SatI(Fnu4HI)	37	C(285)
	RP: CGGCCCTTTGGAACCCTT					
rs293795	FP: GCGCTAAGGATGGTTTTATC	58	309	-	-	-
	RP: CCTCTGTTCTGTCTTCCCAT					

*FP, Forward primer; RP, Reverse primer.
